# Investigation of Pharmacological Mechanisms and Active Ingredients of *Cichorium intybus* L. in Alleviating Renal Urate Deposition via lncRNA H19/miR-21-3p Regulation to Enhance ABCG2 Expression

**DOI:** 10.3390/ijms26167892

**Published:** 2025-08-15

**Authors:** Xiaoye An, Yi Xu, Qiuyue Mao, Chengjin Lu, Xiaoyang Yin, Siying Chen, Bing Zhang, Zhijian Lin, Yu Wang

**Affiliations:** School of Chinese Materia Medica, Beijing University of Chinese Medicine, Beijing 102488, China; 20210935096@bucm.edu.cn (X.A.); 20220941491@bucm.edu.cn (Y.X.); 20190935155@bucm.edu.cn (Q.M.); 20230935117@bucm.edu.cn (C.L.); 20220935209@bucm.edu.cn (X.Y.); siyingchen@bucm.edu.cn (S.C.); zhangb@bucm.edu.cn (B.Z.)

**Keywords:** renal urate deposition, ABCG2, lncRNA H19/miR-21-3p, *Cichorium intybus* L., pharmacological mechanisms, active ingredients

## Abstract

Renal urate deposition is a pathological inflammatory condition characterized by the accumulation of urate crystals in the kidneys, resulting from uric acid supersaturation. *Cichorium intybus* L. (chicory) is a traditional medicinal herb recognized for its efficacy in treating hyperuricemia and gout; however, its effectiveness and underlying mechanisms in mitigating renal urate deposition remain inadequately understood. This study investigates the role of the ATP-binding cassette sub-family G member 2 (ABCG2) transporter and the lncRNA H19/miR-21-3p in renal urate deposition, while also validating the therapeutic effects and mechanisms of chicory extract. Renal urate deposition was induced in rats through the administration of potassium oxonate, adenine, yeast extract, and lipopolysaccharide. The levels of serum uric acid (SUA), urate deposition, inflammation, renal function, and histological changes were analyzed. Dual-luciferase assays, reverse transcription quantitative polymerase chain reaction (RT-qPCR), and immunohistochemistry were utilized to elucidate the relationship among ABCG2, lncRNA H19, and miR-21-3p. The chemical composition and active ingredients of chicory were analyzed using UPLC-LTQ-Orbitrap-MS, along with molecular docking and cell experiments. In rats with renal urate deposition, serum UA levels were elevated, renal UA excretion was reduced, and levels of low inflammatory factors, such as interleukin-6 (IL-6), tumor necrosis factor-alpha (TNF-α), and hypersensitivity C-reactive protein (hs-CRP), were increased. Additionally, significant renal tissue damage accompanied the urate deposition. Notably, these abnormalities were substantially reversed following treatment with chicory extract. A dual-luciferase reporter assay confirmed the regulatory relationships among miR-21-3p, lncRNA H19, and ABCG2. Immunohistochemical analysis and RT-qPCR demonstrated a significant upregulation of miR-21-3p expression, alongside a downregulation of lncRNA H19, *ABCG2* mRNA, and ABCG2 expression in the kidney tissue of rats with renal urate deposition. Chicory extract may exert its inhibitory effect on renal urate deposition by regulating the lncRNA H19/miR-21-3p axis to enhance ABCG2 expression. Furthermore, UPLC-LTQ-Orbitrap-MS identified 69 components in the chicory extract, including scopoletin, quercetin-3-*O*-*β*-D-glucuronide, 11*β*,13-dihydrolactucopicrin, and kaempferol-3-*O*-*β*-D-glucuronide, which were absorbed into the blood of both normal rats and those with renal urate deposition. Molecular docking and cell experiment further validated the effective regulation of 11*β*,13-dihydrolactucopicrin in ABCG2 and the lncRNA H19/miR-21-3p axis. The downregulation of ABCG2, mediated by the lncRNA H19/miR-21-3p axis, may represent a critical pathogenic mechanism in renal urate deposition. Chicory alleviates this deposition by modulating the lncRNA H19/miR-21-3p axis to enhance ABCG2 expression, potentially through its component, 11β,13-dihydrolactucopicrin, thereby revealing novel therapeutic insights for renal urate deposition.

## 1. Introduction

Uric acid (UA) is the final product of human purine metabolism. Disruptions in purine metabolism and UA excretion can elevate blood UA levels [1,2,3]. When blood UA concentrations surpass the solubility threshold, crystalline urate deposits may form in tissues or joints, ultimately leading to conditions such as hyperuricemia nephropathy or gouty arthritis [4,5]. Among these, hyperuricemia nephropathy is particularly noteworthy, as approximately 75% of UA elimination occurs through renal excretion [6,7]. Several UA transporters in the kidney play vital roles in UA excretion by either reabsorbing or secreting it, including urate transporter 1 (URAT1), glucose transporter 9 (GLUT9), organic anion transporters 1 and 3 (OAT1, OAT3), and ABCG2 [8,9,10]. Of particular concern is the accumulation of urate deposits, which can injure the kidneys and subsequently impede UA excretion. This results in a detrimental cycle of ongoing UA retention and kidney injury, suggesting that preventing urate deposition in the kidneys could effectively disrupt this vicious cycle. However, merely reducing blood UA levels is insufficient to prevent urate deposition, as evidenced by the limited efficacy of uricosuric agents in treating UA nephropathy [11,12]. In addition to elevated blood UA levels, our previous research indicates that low-grade inflammation also plays a crucial role in the formation of renal urate deposits [13]. Studies reporting hyperuricemia often note the presence of persistent, chronic, low-grade inflammation, which supports our opinion [14].

Recent reports indicate that ABCG2, the transporter responsible for UA excretion, is also closely associated with inflammatory reactions [15]. For instance, ABCG2 is involved in the mechanism of hyperuricemia-induced renal inflammation in a dependent manner [16]. We hypothesize that ABCG2 may serve as a promising target for interventions aimed at reducing urate deposition in the kidneys. ABCG2 belongs to the subfamily G of the ABC efflux transporter superfamily and comprises one transmembrane domain and one ATP binding domain [16]. The literature indicates that expression and function of ABCG2 can be significantly influenced by various factors, including self-structural effects, single nucleotide polymorphisms, and a variety of transcription factors and hormones [17,18]. Among these factors, non-coding RNAs (ncRNAs), which are functional RNA molecules that do not code for proteins, have emerged as crucial regulators of coding gene transcription levels. This enables the rapid modulation of disease occurrence and progression [19]. MicroRNAs (miRNAs), approximately 20 to 23 nucleotides in length, and long non-coding RNAs (lncRNAs), exceeding 200 nucleotides, represent two prevalent types of ncRNAs [20,21,22]. Studies have shown that miRNAs and lncRNAs are intricately involved in the pathogenesis of abnormal UA metabolism. Specifically, miRNAs can regulate the expression of urate transporter genes. For instance, miR-34a targets SLC22A12 mRNA to inhibit URAT1 [23,24,25]. LncRNAs can function as competitive endogenous RNA (ceRNA) sponges, sequestering miRNAs to modulate the expression of messenger RNA (mRNA) in disease states [26,27]. Notably, a strong connection has been established between lncRNA H19 and the onset of hyperuricemia [28]. Our study observed a significant upregulation of miR-21-3p in rats with renal urate deposition. Through bioinformatic predictive analysis, we identified a potential interaction between miR-21-3p and the binding site of lncRNA H19 and ABCG2. Consequently, the primary aim of this study was to elucidate the relationship between lncRNA H19, miR-21-3p, and ABCG2 using biomedical approaches, including vector construction and dual-luciferase reporter assays.

*Cichorium intybus* L. (chicory), a member of the *Asteraceae* family, is an herb utilized for both medicinal and culinary purposes, with a history tracing back to the eleventh century [29,30]. Its young leaves are commonly consumed in salads and serve as a substitute for coffee. The roots, leaves, and seeds of chicory have been employed in traditional Chinese medicine. According to records from the Chinese Pharmacopoeia, chicory is reputed to purify the liver and gallbladder, strengthen the stomach, and exhibit diuretic and anti-inflammatory properties [31]. Contemporary studies have further demonstrated its therapeutic benefits, including the reduction of SUA levels and the alleviation of inflammatory responses [32,33,34]. Consequently, the following question arises: Does chicory have an ameliorating effect on renal urate deposition? Our previous study revealed that chicory can regulate ABCG2 [35]; however, the underlying mechanism remains unclear. Therefore, the second objective of this study is to determine whether chicory improves renal urate deposition and to elucidate its molecular mechanisms.

## 2. Results

### 2.1. Chicory Ameliorates Renal Urate Deposition in Rats

**Figure 1:**
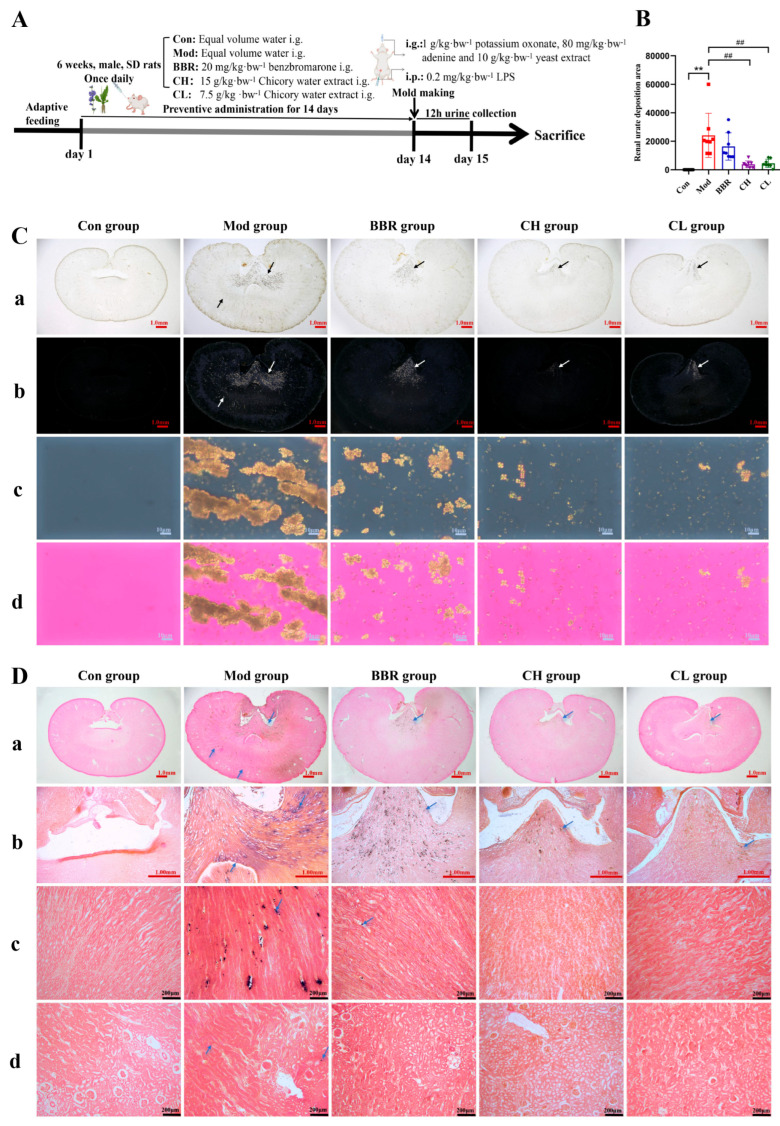
Effects of chicory on renal urate crystal deposition. (**A**) Experimental design scheme. (**B**) Area of renal urate deposition (*n* = 8). (**C**) Observation of renal urate deposition under polarized light (scale bar = 1.00 mm/1.00 mm/10 μm/10 μm, from top to bottom). (**a**) Macroscopic view of the kidney; (**b**,**c**) dark field imaging under polarized light; (**d**) bright field imaging under polarized light. (**D**) Gomori silver hexamine-stained sections of kidney tissue (scale bar = 1.00 mm/1.00 mm/200 μm/200 μm, from top to bottom). (**a**) Macroscopic view of the kidney; (**b**) renal pelvis; (**c**) renal medulla; (**d**) renal cortex. Note: The black/white/blue arrows in the picture point to the urate crystals. ** *p* < 0.01, compared to the Con group. ^##^ *p* < 0.01, compared with the Mod group.

The successful induction of renal urate deposition in rats, following the administration of potassium oxonate, adenine, yeast extract, and lipopolysaccharide (LPS), was unequivocally demonstrated through polarized light microscopy and Gomori silver hexamine staining (Figure 1A,C,D). The observed urate crystals align with the findings reported in reference [36]. Notably, these urate crystals were predominantly localized within the renal pelvis, renal medulla, and renal cortex.

Furthermore, statistical analysis of the deposition areas across the different groups revealed a significant increase in the deposition area within the Mod group compared to the Con group. However, it was observed that the increased deposition area was markedly decreased in both the CH and CL groups. No statistically significant differences were observed in the BBR group (Figure 1B). These findings suggest that chicory significantly reduces renal urate deposition.

### 2.2. Chicory Improved Kidney Dysfunction and Attenuated Renal Histopathologic Injury in Rats with Renal Urate Deposition

**Figure 2:**
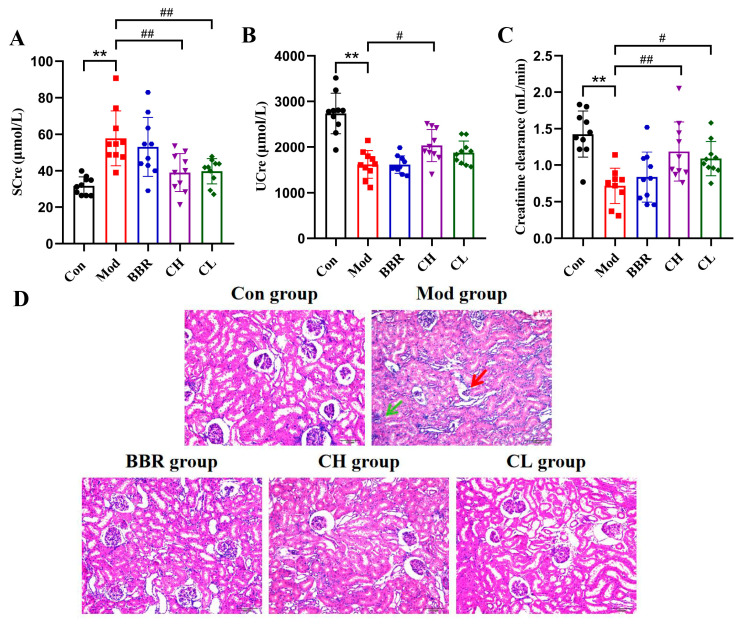
Effects of chicory on renal injury induced by urate deposition. (**A**) Serum creatinine level (SCre, *n* = 10). (**B**) Urine creatinine level (UCre, *n* = 10). (**C**) Creatinine clearance level (*n* = 10). (**D**) H&E-stained sections of kidney tissue (scale bar = 100 μm). The red arrow indicates glomerular atrophy, while the green arrow indicates inflammatory cell infiltration. ** *p* < 0.01, compared to the Con group. ^#^ *p* < 0.05, ^##^ *p* < 0.01, compared with the Mod group.

Serum creatinine (SCre) and urine creatinine (UCre), along with the creatinine clearance rate, are crucial indicators of renal dysfunction, providing valuable insights into glomerular filtration function in clinical practice [37,38]. As anticipated, the Mod group exhibited significantly elevated SCre levels and decreased UCre levels, along with reduced creatinine clearance rates compared to the Con group. In contrast, treatment with chicory extract significantly decreased SCre levels while increasing UCre levels and creatinine clearance rates. No significant differences were observed between the BBR group (Figure 2A–C).

To further investigate the effects of chicory extract on urate deposition-induced renal injury, kidney sections were stained with hematoxylin and eosin (H&E). The staining results revealed more severe glomerular atrophy and inflammatory cell infiltration in the Mod group. In contrast, significantly improved pathological changes were observed in the kidneys of the CH and CL groups (Figure 2D), suggesting a potential renoprotective role of chicory extract.

### 2.3. Chicory Reduced SUA Levels and Attenuated Low-Grade Renal Inflammation in Rats with Renal Urate Deposition

**Figure 3:**
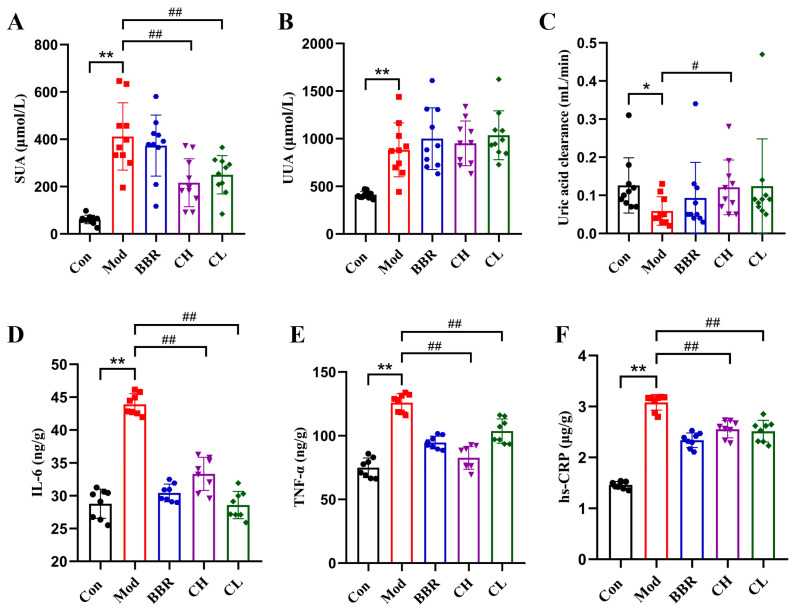
Effects of Chicory on UA levels and renal inflammation. (**A**) Serum uric acid level (SUA, *n* = 10). (**B**) Urine uric acid level (UUA, *n* = 10). (**C**) Uric acid clearance level (*n* = 10). (**D**–**F**) Levels of IL-6, TNF-α, and hs-CRP in kidney tissues (*n* = 8). * *p* < 0.05, ** *p* < 0.01, compared to the Con group. ^#^ *p* < 0.05, ^##^ *p* < 0.01, compared with the Mod group.

The Mod group exhibited increased SUA and urine uric acid (UUA) levels, alongside a decreased uric acid clearance rate compared to the Con group. Conversely, SUA levels were markedly reduced in both the CH and CL groups when compared to the Mod group. Furthermore, the uric acid clearance rate was notably elevated in the CH group, with a positive trend observed in the CL group. No significant differences were noted in the BBR group (Figure 3A–C).

Low-grade inflammation is indicated by the overproduction of acute phase proteins such as hypersensitivity C-reactive protein (hs-CRP) and pro-inflammatory cytokines like interleukin-6 (IL-6) and tumor necrosis factor-alpha (TNF-α) [39,40]. The results demonstrated that the levels of IL-6, TNF-α and hs-CRP were markedly elevated in the Mod group compared to the Con group. However, the CH, CL, and BBR groups showed significantly reduced levels of IL-6, TNF-α and hs-CRP (Figure 3D–F). These findings indicate that chicory has a significant effect on reducing UA levels and alleviating low-grade renal inflammation.

### 2.4. Chicory Increased ABCG2 Regulated by lncRNA H19/miR-21-3p in Rats with Renal Urate Deposition

#### 2.4.1. Analysis of Targeting Regulatory Relations Between miR-21-3p, lncRNA H19, and ABCG2

**Figure 4:**
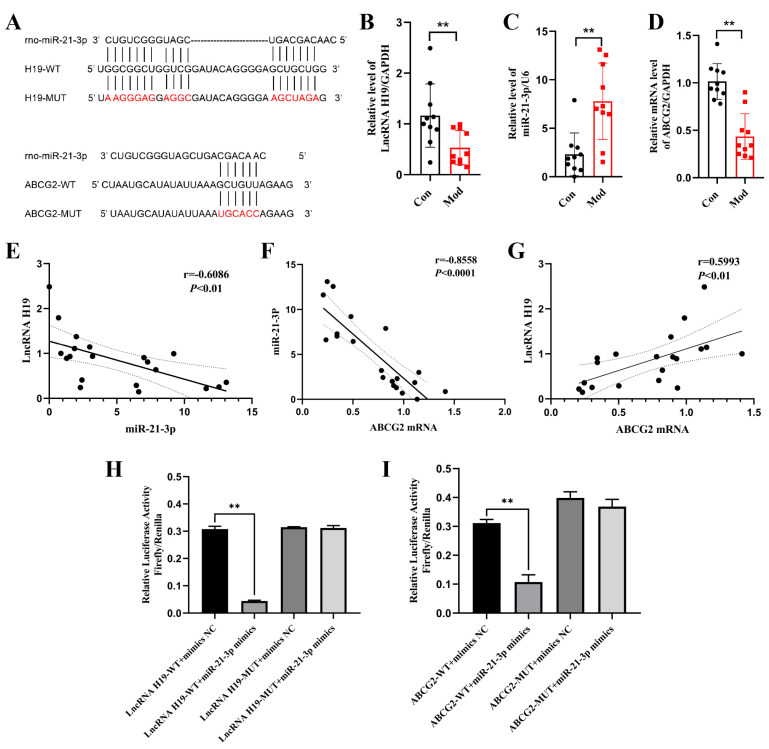
Analysis of the lncRNA H19/miR-21-3p/ABCG2 in renal urate deposition. (**A**) Alignment of the sequences of lncRNA H19 and ABCG2 with miR-21-3p, including both the wild type (WT) and mutant (MUT) sequence. Schematic illustration of the presumed target site for lncRNA H19 and ABCG2 within miR-21-3p. (**B**–**D**) mRNA levels of lncRNA H19, miR-21-3p, and ABCG2 in kidney tissue (*n* = 10). (**E**–**G**) Correlation analysis between lncRNA H19 and miR-21-3p, miR-21-3p, and ABCG2, as well as lncRNA H19 and ABCG2 levels (*n* = 20). (**H**) Direct binding between lncRNA H19 and miR-21-3p was confirmed (*n* = 3). (**I**) miR-21-3p directly regulates the expression of ABCG2 (*n* = 3). ** *p* < 0.01, compared to the Con group or the lncRNA H19/ABCG2-WT + mimics NC group.

To explore the regulatory interactions among miR-21-3p, lncRNA H19, and ABCG2, we performed predictive analyses using various tools: BiBiServ2 for binding site prediction between lncRNA H19 and miR-21-3p, lnclocater and LncATLAS for subcellular localization of lncRNA H19, and TargetScan for binding site prediction between miR-21-3p and ABCG2. Our findings suggest that lncRNA H19 may function as a ceRNA for miR-21-3p, sharing potential binding sites (Figure 4A). Additionally, ABCG2 is identified as a target gene of miR-21-3p, with binding sites located in its 3′-UTR (Figure 4A).

To further validate our findings, we conducted animal experiments and luciferase reporter assays. The animal experiments demonstrated that, in rats with renal urate deposition, the expression of miR-21-3p was significantly upregulated, whereas the expression of both lncRNA H19 and ABCG2 was significantly downregulated. Notably, a strong negative correlation was observed between the expression levels of miR-21-3p and lncRNA H19 (*r* = −0.6086), as well as between miR-21-3p and ABCG2 (*r* = −0.8558). Conversely, a strong positive correlation was found between the expression levels of lncRNA H19 and ABCG2 (*r* = 0.5993) (Figure 4B–G).

The luciferase reporter assay revealed that the miR-21-3p mimic significantly suppressed the luciferase activity in cells harboring the wild-type lncRNA H19/ABCG2 constructs (WT-lncRNA H19/ABCG2), whereas it had no significant inhibitory effect on the luciferase activity of cells with the mutant lncRNA H19/ABCG2 constructs (MUT-lncRNA H19/ABCG2) (Figure 4H,I).

Upon integrating the experimental data, our findings indicate that the regulatory interplay among miR-21-3p, lncRNA H19, and ABCG2 could serve as a potential molecular mechanism underlying the progression of renal urate deposition.

#### 2.4.2. Chicory Promotes ABCG2 Expression Regulated by lncRNA H19/miR-21-3p

To elucidate the potential molecular mechanism underlying the treatment of renal urate deposition with chicory extract, we examined the expression levels of lncRNA H19, miR-21-3p, *ABCG2* mRNA, and ABCG2 protein in kidney tissue. The Qsep100^TM^ fully automated nucleic acid and protein analysis system demonstrated that all sample total RNA had RQN values exceeding 7, confirming their high purity and integrity for subsequent analysis (Figure S1).

**Figure 5:**
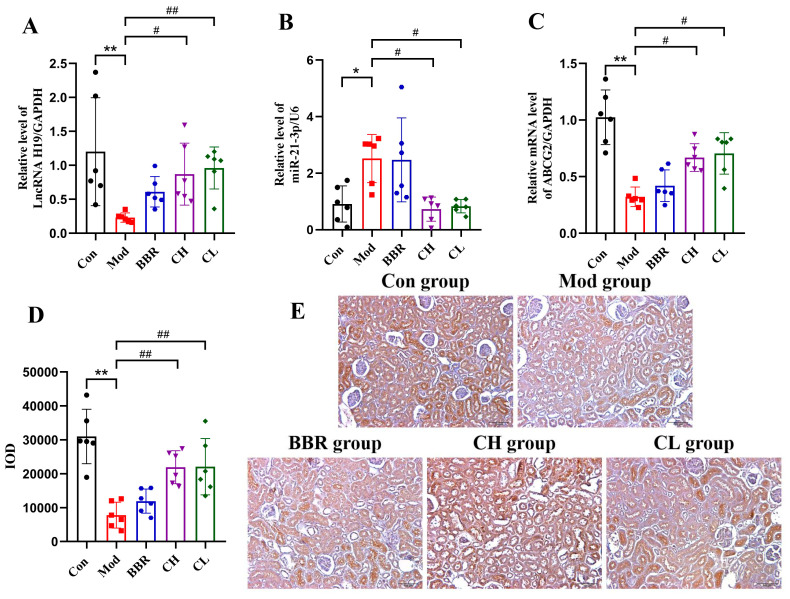
Effects of chicory on ABCG2 expression regulated by the lncRNA H19/miR-21-3p axis in kidney tissue. (**A**–**C**) Levels of lncRNA H19, miR-21-3p, and *ABCG2* mRNA in kidney tissue (*n* = 6). (**D**) Expression level of ABCG2 protein in kidney tissue (*n* = 6). (**E**) Immunohistochemical staining of ABCG2 in kidney tissue (scale bar = 100 μm). * *p* < 0.05, ** *p* < 0.01, compared to the Con group. ^#^ *p* < 0.05, ^##^ *p* < 0.01, compared to the Mod group.

RT-qPCR detection revealed that in the Mod group, the expression of miR-21-3p was significantly upregulated, while the expressions of lncRNA H19 and ABCG2 mRNA were significantly downregulated in kidney tissue (Figure 5A–C). Immunohistochemical staining further confirmed a significant downregulation of ABCG2 protein in the kidney tissue of the Mod group (Figure 5D,E). Conversely, in the CH and CL groups, the expressions of lncRNA H19, *ABCG2* mRNA, and ABCG2 protein were increased, while the expression of miR-21-3p was reduced. No significant differences were observed in the BBR group.

These results, along with the observed improvements in both the deposition area and low-grade renal inflammation in the CH and CL groups, indicate that chicory extract may exert its inhibitory effect on renal urate deposition by promoting ABCG2 expression through the regulation of the lncRNA H19/miR-21-3p axis.

### 2.5. Analysis of Active Ingredients in Chicory Extract to Alleviate Renal Urate Deposition

A total of 69 compounds in chicory extract were identified using UPLC-LTQ Orbitrap MS. The details regarding the identified peaks, including retention time, molecular formula, ion type, detected mass, mass error, and fragment ions, are summarized in Figure S2. Furthermore, we conducted an analysis of the constituents absorbed into blood of both normal rats and rats with renal urate deposition following the administration of chicory extract.

The results revealed that a total of 13 prototype components were identified in the plasma of normal rats after chicory extract administration, while only 9 prototype components were identified in the plasma of rats with renal urate deposition (Figure S3 and Table 1). Notably, four of these components were confirmed through chemical standard samples: scopoletin, quercetin-3-*O*-*β*-D-glucuronide, 11*β*,13-dihydrolactucopicrin, and kaempferol-3-*O*-*β*-D-glucuronide.

### 2.6. Analysis of Active Ingredients in Chicory Extract Targeting ABCG2 and the lncRNA H19/miR-21-3p Axis

#### Cell Experiments

We conducted molecular docking analyses to assess the binding capabilities of four active ingredients with ABCG2, as identified from serum samples. The docking results revealed binding energies ranging from −7.5 to −11.2 kcal/mol, suggesting that all four ingredients exhibit favorable binding activities with the target protein ABCG2 (Figure S4, Tables S1–S3).

**Figure 6:**
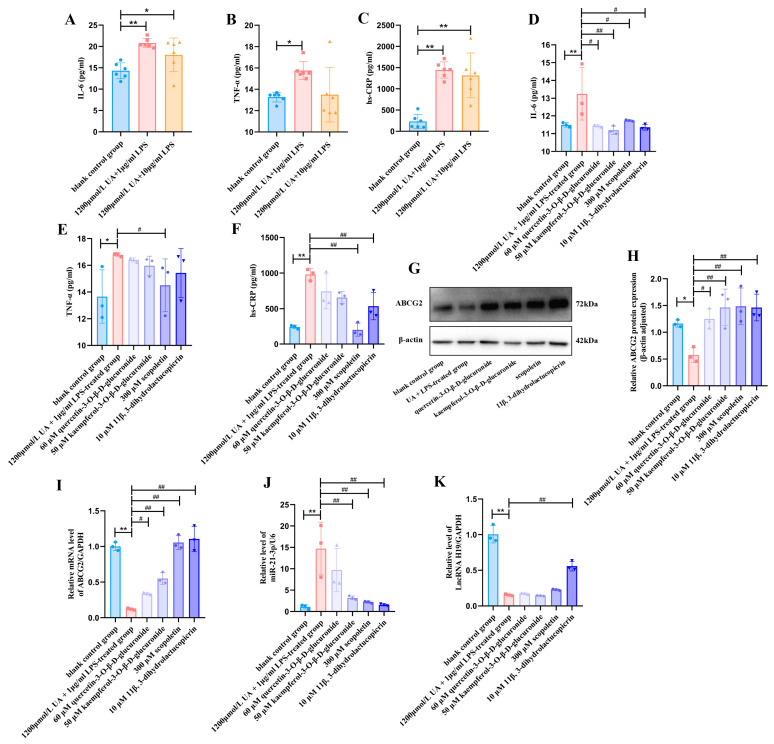
Analysis of active ingredients in chicory extract targeting the lncRNA H19/miR-21-3p/ABCG2 in vitro. (**A**–**C**) Levels of IL-6, TNF-α, and hs-CRP in the NRK-52E cells treated with 1200 μmol/L UA and 1 μg/mL LPS (*n* = 6). (**D**–**F**) Levels of IL-6, TNF-α, and hs-CRP in the supernatant of NRK-52E cells (*n* = 3). (**G**,**H**) Representative images and semi-quantification analysis of ABCG2 protein expression in NRK-52E cells (*n* = 3). (**I**–**K**) mRNA levels of lncRNA H19, miR-21-3p, and ABCG2 in NRK-52E cells (*n* = 3). where, * *p* < 0.05, ** *p* < 0.01, compared to the blank control group. ^#^ *p* < 0.05, ^##^ *p* < 0.01, compared with the UA and LPS-treated group.

To create Normal rat kidney-52 epithelial (NRK-52E) cells with UA and LPS, we performed a concentration selection based on cell viability and the levels of IL-6, TNF-α, and hs-CRP in the NRK-52E cell supernatant. Our results indicated that the levels of IL-6, TNF-α, and hs-CRP were significantly elevated in cells treated with 1200 μmol/L UA + 1 μg/mL LPS, compared to the blank control group (Figure 6A–C). Based on these findings, we selected concentrations of 1200 μmol/L UA and 1 μg/mL LPS for subsequent experiments. Similarly, based on the analysis of active ingredients in chicory extract, we chose 60 μM quercetin-3-*O*-*β*-D-glucuronide, 50 μM kaempferol-3-*O*-*β*-D-glucuronide, 10 μM 11*β*,13-dihydrolactucopicrin, and 300 μM scopoletin for subsequent experiments (Figure S5).

The results demonstrated that IL-6 levels were significantly suppressed in the groups treated with the four ingredients compared to the UA and LPS-treated group (Figure 6D–F). TNF-α levels were notably inhibited in the scopoletin-treated group, while hs-CRP levels were significantly decreased in both the scopoletin and 11*β*,13-dihydrolactucopicrin-tretaed group. Western blot analysis revealed that ABCG2 protein expression was upregulated in the groups treated with the four ingredients, compared to the UA and LPS-treated group (Figure 6G,H). Additionally, RT-qPCR analysis showed that ABCG2 mRNA expression was also elevated in these groups. Among the ingredients, lncRNA H19 expression was uniquely and significantly upregulated in the 11*β*,13-dihydrolactucopicrin-treated group. Conversely, miR-21-3p expression was markedly downregulated in the kaempferol-3-*O*-*β*-D-glucuronide, 11*β*,13-dihydrolactucopicrin, and scopoletin-treated groups compared to the UA and LPS-treated group (Figure 6I–K).

## 3. Discussion

### 3.1. Establishment of In Vivo and In Vitro Models for Renal Urate Deposition

Reliable animal models are fundamental for investigating the pathogenesis of urate renal deposition and for drug development. The deposition of urate crystals serves not only as the gold standard for diagnosing gouty arthritis but also as a key pathological indicator of uric acid nephropathy. Accurately simulating this process is crucial for understanding the disease [41]. However, most current animal models of uric acid nephropathy primarily focus on renal injury (e.g., biomarkers of renal dysfunction) while neglecting the core pathological aspect of urate deposition [42]. For instance, conventional modeling methods using potassium oxonate or adenine can elevate serum uric acid levels and induce renal dysfunction but fail to achieve stable urate crystal formation in the kidneys [43]. Although urate oxidase knockout animal models maintain stable hyperuricemia, they are often accompanied by developmental abnormalities and high mortality rates, which can interfere with renal pathological assessments [44].

Studies have demonstrated that hyperuricemia serves as the biochemical foundation for renal urate deposition, however, merely reducing serum uric acid levels is insufficient to prevent this deposition [11,12]. In addition to elevated SUA levels, mild inflammation plays a pivotal role in the formation of renal urate deposits. Research indicates that inhibiting inflammation can directly ameliorate renal injury caused by urate accumulation [8,45].

Based on these findings, our study utilized potassium oxonate, adenine, and yeast extract (recognized inducers of elevated UA levels), along with low-dose lipopolysaccharide (LPS, an inducer of mild inflammation), to establish a rat model that comprehensively reflects urate renal deposition under conditions of hyperuricemia accompanied by mild inflammation [13]. Despite its relatively complex operational requirements, this model effectively simulates the key phenotype of urate crystals, facilitating the successful observation of distinct urate deposition in the kidneys of experimental rats. Furthermore, it validates the equally important roles of hyperuricemia and mild inflammation in the formation of urate deposits.

Currently, there is a lack of reliable in vitro cellular models for studying urate deposition. Our preliminary attempts to directly intervene with NRK-52E cells using monosodium urate crystals to mimic the pathological state of urate deposition significantly affected cellular status, leading to decreased cell viability and even cell death, which greatly hindered our investigation into the pathological mechanisms of urate deposition. Drawing from our experience with animal modeling, we found that using UA at a crystallization-saturated concentration in combination with low-dose LPS could stably induce renal urate deposition accompanied by characteristic inflammatory responses. Consequently, we optimized and established an in vitro model by stimulating NRK-52E cells with 1200 μmol/L UA and 1 μg/mL LPS. The results demonstrated a significant downregulation of the urate transporter ABCG2 protein expression and the substantial release of IL-6-dominated inflammatory cytokines. Although the in vitro system cannot fully replicate the crystal deposition process, it successfully recapitulates the key molecular events in urate deposition (urate transport disorders + specific inflammatory activation), providing a reproducible cellular-level platform for mechanistic studies.

In conclusion, these in vitro and in vivo models are invaluable research tools for investigating the pathological mechanisms of urate renal deposition.

### 3.2. The Pharmacological Effect and Active Ingredients of Chicory in Renal Urate Deposition

Previous studies have established the anti-gout and anti-hyperuricemia properties of chicory [33,34]. In the present study, we found that chicory extract can also reduce renal urate deposition in experimental rats. Given the critical roles of UA excretion and inflammatory responses in the pathogenesis of renal urate deposition, we assessed the reduction of renal injury, the enhancement of both creatinine clearance rate and uric acid clearance rate, as well as the downregulation of IL-6, TNF-α, and hs-CRP expression in rats treated with chicory extract. These observations suggest that chicory exerts a dual therapeutic effect by promoting UA excretion and ameliorating the low-grade inflammatory state within the kidney. Our study identifies ABCG2 as a promising target for elucidating the mechanisms underlying renal urate deposition.

Additionally, miR-21-3p and lncRNA H19 may be involved in the regulation of ABCG2 expression. Through bioinformatic analysis and a dual-luciferase reporter assay, we determined the binding sites among miR-21-3p, lncRNA H19, and ABCG2. Subsequently, our observations revealed significantly upregulated expression of miR-21-3p and significantly downregulated expression of lncRNA H19 and ABCG2 mRNA in rats with renal urate deposition. These results confirm the regulatory relationships among miR-21-3p, lncRNA H19, and ABCG2 mRNA, indicating that lncRNA H19 competes with miR-21-3p, thereby attenuating the regulatory effect of miR-21-3p on ABCG2 mRNA.

Concurrently, we observed that chicory extract effectively increased the expression levels of lncRNA H19, ABCG2 mRNA, and ABCG2 protein, while simultaneously decreasing the expression of miR-21-3p in the kidneys of rats with renal urate deposition. These findings indicate that chicory extract upregulates ABCG2, which subsequently enhances UA excretion and mitigates the low-grade inflammatory response in the kidney, thereby suppressing renal urate deposition. This effect may be mediated through the modulation of ABCG2 expression by the lncRNA H19/miR-21-3p axis.

Generally, the constituents of herbal medicine that are absorbed into the blood following oral administration are widely regards as the pharmacodynamically active components [46]. In our study, we utilized UPLC-LTQ-Orbitrap-MS to identify 69 components in chicory extract and confirmed the absorption of four prototype ingredients into the blood of rats with renal deposition. The four chemical ingredients are quercetin-3-*O*-*β*-D-glucuronide, kaempferol-3-*O*-*β*-D-glucuronide, scopoletin, and 11*β*,13-dihydrolactucopicrin. Quercetin-3-*O*-*β*-D-glucuronide, a glucuronide conjugate of quercetin, has been reported to exhibit anti-inflammatory properties in LPS-stimulated macrophages [47]. Kaempferol-3-*O*-*β*-D-glucuronide, a plant-derived flavonoid glycoside, demonstrates antioxidant and anti-inflammatory activities [48,49,50]. Scopoletin, a phenolic coumarin derived from various medicinal or edible plants, plays a role in numerous pharmacological functions, including anti-cancer, anti-diabetic, anti-inflammatory, cardioprotective, and hepatoprotective activities [51,52]. 11*β*,13-dihydrolactucopicrin, a sesquiterpenoid lactone compound that serves as the primary bitter constituent of chicory roots [53], has shown neuroprotective and anti-inflammatory effects in pharmacological studies [54]. Notably, all these compounds exhibit anti-inflammatory properties in our cell experiments.

Molecular docking results indicate that these four ingredients have favorable binding activities with ABCG2. All four compounds were found to dock into the active pocket of the target protein ABCG2, primarily forming interactions with the amino acid residues through hydrogen bonds and hydrophobic effects. Among them, 11*β*,13-dihydrolactucopicrin exhibited the lowest binding energy with the ABCG2 protein, suggesting its potential as a key bioactive compound for modulating ABCG2 expression. To gain a deeper and more comprehensive understanding of the active ingredients in chicory that inhibit renal urate deposition, we examined the expression of the lncRNA H19/miR-21-3p/ABCG2 in NRK-52E cells following treatment with each of these four ingredients individually. The results indicate that all four ingredients significantly increased ABCG2 mRNA and protein expressions, further emphasizing the important role of ABCG2 in both the pathological process of renal urate deposition and its treatment. Among these four ingredients, 11*β*,13-dihydrolactucopicrin significantly promoted lncRNA H19 repression and suppressed miR-21-3p expression in NRK-52E cells, aligning with the results observed in rats treated with chicory extract. This suggests that 11*β*,13-dihydrolactucopicrin may be a potential component of chicory in alleviating renal urate deposition. Kaempferol-3-*O*-*β*-D-glucuronide and scopoletin exhibited significant inhibition of miR-21-3p expression, while having no effect on lncRNA H19 expression. Conversely, quercetin-3-*O*-*β*-D-glucuronide showed no effect on either miR-21-3p or lncRNA H19. These differing results may be associated with the complex regulatory relationships within the ceRNA network, where a single mRNA can be regulated by multiple ncRNAs [55,56]. Further studies are needed to explore the underlying mechanisms of chicory’s effect on renal urate deposition.

## 4. Materials and Methods

### 4.1. Preparation of Chicory Solution

The aboveground parts of chicory utilized in this study were sourced from Xinjiang, China. Chicory was grinded, weighed, and boiled in water for one hour, repeating this process twice to prepare the extracts. The filtered solution was then concentrated using a rotary evaporator and subsequently diluted with purified water to achieve various volumes.

### 4.2. Animal Experiments

#### 4.2.1. Drug Administration and Sample Collection

SPF Sprague–Dawley rats (male, healthy) weighing approximately (180 ± 10) g were purchased from SPF (Beijing) BIOTECHNOLOGY Co., Ltd. (Beijing, China). The license number for the experimental animals is SCXK (Jing) 2019-0010. The rats were housed at 20~25 °C, 35–75% humidity. The experiment was conducted following a 12-h light/dark cycle, with the animals having free access to food and water, and an adaptive feeding period of three days. All procedures involving animals were approved by the Animal Care and Ethics Committee of Beijing University of Chinese Medicine (BUCM-2023050502-2073).

Fifty rats were randomly divided into five groups based on body weight: the control group (Con, *n* = 10), the model group (Mod, *n* = 10), the benzbromarone-treated group (BBR, *n* = 10, Excella GmbH & Co.KG, Renningen, Germany), the high-dosage chicory-treated group (CH group, *n* = 10), and the low-dosage chicory-treated group (CL group, *n* = 10). Except for the Con group, all groups were administered potassium oxonate (1 g/kg body weight, Shanghai Yuanye Bio-Technology Co., Ltd., Shanghai, China), adenine (80 mg/kg body weight, Sigma-Aldrich, Schnelldorf, Germany), and yeast extract (10 g/kg body weight, Thermo Fisher Scientific, Waltham, MA, USA) via gavage, and received a single intraperitoneal injection of Lipopolysaccharide (LPS, 0.2 mg/kg body weight, Sigma-Aldrich, Schnelldorf, Germany) [13,57]. Rats in the Con group were administered a 0.5% CMC-Na solution via gavage and an intraperitoneal injection of physiological saline. Rats in the chicory and benzbromarone groups received the extract or drug for 14 days prior to modeling, followed by daily administration for 14 days. The administration of chicory was determined based on the clinical dose conversion of the Chinese Pharmacopoeia (2025 edition) and the preliminary research basis of the research group, that is, the low dose of chicory was 7.5 g/kg/d (human clinical equivalent dose), and the high dose was 15 g/kg/d (twice the human clinical equivalent dose). During the experiment, the Con and Mod groups were intragastrically administered an equal volume of purified water. Blood samples, urine samples, and kidney tissues were collected according to the experimental design. The levels of uric acid (UA) and creatinine (Cre) in serum and urine were measured following the kit instructions. The levels of IL-6, TNF-α, and hs-CRP were measured using rat ELISA kits (Jiangsu Meimian Industrial Co., Ltd., Changzhou, China).

#### 4.2.2. Histological Analysis

This section includes hematoxylin and eosin (H&E) staining, polarized light observation, and Gomori silver hexamine staining.

Kidney tissues were fixed in anhydrous ethanol, and subsequently embedded in paraffin. Sections measuring 3 μm were stained with H&E (Solarbio, Beijing, China). Images were acquired using a microscope (Nikon, Tokyo, Japan) and analyzed with an imaging system (Nikon, Tokyo, Japan).

For polarized light observation, kidney sections were examined with a microscope (Nikon, Tokyo, Japan). The sections were stained with Gomori silver hexamine stain (Solarbio, Beijing, China), and images were similarly acquired and analyzed using the Nikon imaging system.

#### 4.2.3. Immunohistochemical Analysis

Immunohistochemical analysis of kidney sections was performed by heating the sections in an EDTA antigen retrieval solution (ZSGB-Bio, Shanghai, China) to facilitate antigen retrieval. After blocking endogenous peroxidase activity with 3% H_2_O_2_ and nonspecific binding with goat serum (ZSGB-Bio, Shanghai, China), the sections were incubated overnight at 4 °C with the ABCG2 antibody (1:4000, Abcam, Cambridge, UK). Subsequently, the sections were treated with a universal two-step detection kit (ZSGB-Bio, Shanghai, China) for 30 min at 37 °C, followed by application of a DAB detection kit to visualize positive expression. The sections were then counterstained with hematoxylin. All measurements were conducted using an automated upright microscope system (Olympus, Tokyo, Japan). Five images from each section were randomly captured using a high-speed color CCD camera (Olympus, Tokyo, Japan) and quantified using ImageJ software, version 1.54 (National Institutes of Health, Bethesda, MD, USA).

#### 4.2.4. RT-qPCR Analysis

Total RNA was extracted from tissues using TRIzol (Thermo Fisher Scientific, Waltham, MA, USA), while miRNA was extracted using the Mipure Cell/Tissue miRNA Kit-box 2 (Vazyme, Nanjing, China). The integrity of the total RNA was assessed using an RNA cartridge kit with the Qsep100^TM^ Fully Automated Nucleic Acid and Protein Analysis System (Bioptic Inc., Taiwan, China) (Figure S6). The mRNA and miRNA were reverse-transcribed using the RevertAid First Strand cDNA Synthesis Kit (Thermo Fisher Scientific, Waltham, MA, USA) and the miRNA 1st Strand cDNA Synthesis Kit (Vazyme, Nanjing, China). Quantitative real-time polymerase chain reaction (RT-qPCR) testing was conducted using either the PowerUp^TM^ SYBR^TM^ Green Master Mix (Thermo Fisher Scientific, Waltham, MA, USA) or the miRNA Universal SYBR qPCR Master Mix (Vazyme, Nanjing, China) on the CFX96 Touch Real-Time PCR Detection System (BIO RAD, Hercules, CA, USA). Rat GAPDH and U6 were used as reference genes, with the primers listed in Table 2.

### 4.3. Analysis of the lncRNA H19/miR-21-3p/ABCG2 in Renal Urate Deposition

#### 4.3.1. Prediction of Targeting Regulatory Relations Between miR-21-3p, lncRNA H19, and ABCG2

To predict the binding site between lncRNA H19 and miR-21-3p, we utilized BiBiServ2. We employed lncLocater and LncATLAS to predict the subcellular localization of lncRNA H19. Additionally, TargetScan was used to predict the binding site between miR-21-3p and ABCG2. The details of the database sites are shown in Table 3.

#### 4.3.2. Animal Experiment

The 20 rats were randomly divided into control (*n* = 10) and model (*n* = 10) groups based on body mass. Following the induction of model using potassium oxonate, adenine, yeast extract, and LPS, kidney tissues were collected. The expression levels of lncRNA H19, miR-21-3p, and ABCG2 were quantified using RT-qPCR, and a correlation analysis was subsequently conducted.

#### 4.3.3. Luciferase Reporter Assay

The 293T cells were cultured in DMEM supplemented with 10% fetal bovine serum and 1% penicillin-streptomycin solution and incubated at 37 °C, 5% CO_2_ with 95% air.

Based on the BiBiServ2 database and the Targeted Scan database, we predicted the regions with higher binding scores for lncRNA H19 and miR-21-3p, as well as for miR-21-3p and ABCG2. The fragment of lncRNA H19/ABCG2 containing the miR-21-3p binding sites, along with the corresponding mutants, was subcloned into the pmirGLO dual-luciferase miRNA target expression vector to construct the lncRNA H19/ABCG2-mutated-type (lncRNA H19/ABCG2-MUT) and lncRNA H19/ABCG2-wild-type (lncRNA H19/ABCG2-WT) plasmids. Subsequently, 293T cells were plated onto 24-well plates at a density of 4 × 10^4^ cells per well. After 24 h, the plasmids lncRNA H19/ABCG2-WT or lncRNA H19/ABCG2-MUT were co-transfected into 293T cells along with miR-NC or miR-21-3p mimics using JETPRIM transfection reagent (Polyplus, Illkirch, France). Luciferase activities were measured 48 h post-transfection, following the instructions of the Dual-Luciferase Reporter Assay System Kit (Promega, Madison, WI, USA). This portion of the experiment was entrusted to Hebei Lebo Pharmaceutical Technology Co., Ltd., Shijiazhuang, China.

### 4.4. Active Ingredients Analysis

#### 4.4.1. Sample Preparation

The analysis of active ingredients involves examining the chemical constituents present in chicory extract and the corresponding constituents in the blood.

To prepare the sample, 1 mL of chicory extract was pipetted into a measuring bottle and diluted with methanol to the desired volume. The sample was then filtered through a 0.22 µm membrane prior to injection, and the resulting solution was utilized for UPLC-LTQ-Orbitrap-MS analysis.

Twenty-four rats were randomly assigned to four groups based on their body mass: the blank normal group, the chicory-treated normal group, the blank model group, and the chicory-treated model group, with six rats in each group. The modeling was conducted using potassium oxonate, adenine, yeast extract, and LPS. Rats in the chicory-treated normal group and the chicory-treated model group received an intragastric administration of chicory extract at a dose of 30 g/kg every 12 h for a total of five doses. In contrast, the blank normal group and the blank model group were administered the corresponding volumes of pure water. Orbital blood samples were collected at 0.5, 1, 2, and 4 h after the final administration and placed in heparinized test tubes. These samples were centrifuged at 3500 rpm for 10 min to obtain plasma.

For each group, 41.3 μL of plasma was mixed with three volumes of acetonitrile and vortexed for 3 min. Proteins were removed by centrifugation at 10,000 rpm at 4 °C for 10 min. The supernatant was collected and dried under nitrogen. The samples were then re-dissolved in 100 μL methanol, centrifuged at 15,000 rpm at 4 °C for 5 min, and the supernatant was used for injection analysis.

#### 4.4.2. UPLC-LTQ-Orbitrap-MS Analysis

UPLC analysis was conducted on an UltiMate 3000 system (Thermo Scientific, Waltham, MA, USA) utilizing an ACQUITY UPLC HSS T3 column (2.1 mm × 100 mm, 1.8 µm, Waters, Milford, MA, USA) for chromatographic separation. The mobile phases consisted of solvent A (0.1% formic acid in water) and solvent B (methanol). Gradient elution was applied as follows: 0–3 min, 10% B; 3–15 min, 10–25% B; 15–29 min, 25–40% B; 29–38 min, 40–45% B; 38–50 min, 45–70% B; 50–55 min, 70–100% B; 55–60 min, 100% B; 60–61 min, 100–10% B; 61–65 min, 10% B. Injection volume: 2 μL, column: 30 °C, flow: 0.2 mL/min.

MS analysis was conducted using an LTQ-Orbitrap system (Thermo Fisher Scientific, Waltham, MA, USA) equipped with an electrospray ionization source, operating in both positive and negative ion mode. The ion source temperature was set to 350 °C. The spray voltage was adjusted to 4 kV in positive ion mode and 3 kV in negative ion mode, while the capillary voltage was maintained at 35 V. The sheath gas flow was set to 40.00 arb, and the auxiliary gas flow was 20 arb. Collision-induced dissociation was employed as the fragmentation technique, with a normalized collision energy of 35%. Reference masses were recorded within an m/z range of 100 to 1000. The acquired data were analyzed using Compound Discover, version 3.3 and Xcalibur software, version 4.2 (Thermo Fisher Scientific, Waltham, MA, USA).

### 4.5. Cell Experiments

#### 4.5.1. Cell Culture and Cell Viability Assay

NRK-52E cells were purchased from the Cell Resource Center at the Institute of Basic Medical Sciences, Chinese Academy of Medical Sciences and Peking Union Medical College (CAMS/PUMC) (Beijing, China). The cells were cultured in DMEM(H) medium (Thermo Fisher Scientific, Waltham, MA, USA) supplemented with 5% fetal bovine serum (ExCell Bio, Shanghai, China) and 1% penicillin-streptomycin solution (Thermo Fisher Scientific, Waltham, MA, USA). They were incubated at 37 °C in an atmosphere of 5% CO_2_ and 95% air.

NRK-52E cells in the logarithmic growth phase were inoculated into 96-well plates at a density of 4000 cells per well, with six replicates for each group. After allowing the cells to adhere, they were treated with UA at concentrations of 120, 240, 480, 960, and 1200 μmol/L, as well as with LPS at concentrations of 0.1, 1, 2, 5, and 10 μg/mL. The optimal concentrations of UA and LPS were subsequently determined. Based on preliminary experimental results, the cells were incubated with quercetin-3-*O*-*β*-D-glucuronide at concentrations of 10, 20, 30, 40, and 60 μM, kaempferol-3-*O*-*β*-D-glucuronide at concentrations of 5, 10, 20, 40, and 50 μM, scopoletin at concentrations of 50, 100, 150, 200, and 300 μM, and 11*β*,13-dihydrolactucopicrin at concentrations of 10, 50, 100, 200, and 500 μM. The selection of these chemical components was based on a comprehensive review of the literature, recommended dissolution concentrations for commercial chemical substances, and findings from our preliminary experiments. After 24 h of treatment, the culture medium was removed, and 110 μL of CCK-8 solution (prepared by mixing DMEM(H) and CCK-8 solution in a 10:1 ratio) was added to each well. The plate was then placed in a 37 °C incubator, and color changes were continuously monitored. Once optimal color development achieved, then absorbance was measured the at 450 nm.

#### 4.5.2. Western Blotting Analysis

Total protein extracts from NRK-52E cells were obtained using a cell lysis buffer, separated by SDS-PAGE, and subsequently transferred to PVDF membranes. After blocking with 5% non-fat milk, the membranes were incubated overnight at 4 °C with the ABCG2 primary antibody (1:1000, A17908, ABclonal, Wuhan, China). Following this, the membranes were treated with an HRP-conjugated goat anti-rabbit secondary antibody (1:5000, SA00001-2, Proteintech, Rosemont, IL, USA). We used β-actin (1:5000, 66009-1-Ig, Proteintech, Rosemont, IL, USA) as a control. Bands were visualized using MiniChemi Chemiluminescent Imaging and Analysis System (Beijing Sage Creation, Beijing, China) and quantified with ImageJ software.

#### 4.5.3. RT-qPCR Analysis

The procedure was the same as described in Section 4.2.4.

### 4.6. Statistical Analysis

Statistical analyses were conducted using GraphPad Prism 8.0 software (GraphPad Software Inc., La Jolla, CA, USA). Results are presented as the mean ± standard deviation (SD). The data were first evaluated for normality. For normally distributed data, one-way analysis of variance (ANOVA) was employed for multiple group comparisons. When homogeneity of variance was confirmed, post hoc pairwise comparisons were performed using Dunnett’s test. In cases of unequal variance, Dunnett’s T3 test was applied for between-group comparisons. For non-normally distributed data, the Kruskal–Wallis rank sum test was used for multiple groups comparisons, followed by Dunn’s test for post hoc pairwise comparisons between groups. A *p*-value of less than 0.05 was deemed statistically significant.

## 5. Conclusions

Our study identifies ABCG2 as a crucial target for elucidating the mechanisms underlying renal urate deposition, due to its complex association with both UA excretion and inflammatory responses in the kidney. The downregulation of ABCG2, mediated by the lncRNA H19/miR-21-3p axis, may represent a significant pathogenic mechanism in renal urate deposition. Furthermore, we elucidate the mechanism by which *Cichorium intybus* L. (chicory) alleviates renal urate deposition by modulating the lncRNA H19/miR-21-3p axis to enhance ABCG2 expression, potentially through its component, 11β,13-dihydrolactucopicrin, thereby providing novel therapeutic insights.

## Figures and Tables

**Table 1:** Analysis and identification of prototype components in the plasma of chicory-treated normal rats and rats with renal urate deposition.

Group	No.	Compound	t_R_/min	Formula	Ion Type	Theoretical Molecular Weight (m/z)	Mass Error (ppm)	MS/MS (m/z)
Normal rats	1 **^a^**	quinic acid	1.53	C_7_H_12_O_6_	[M-H]^-^	191.05611	0.26	172.8746, 126.9849, 111.0423, 92.9883, 84.9185
	2	matrine	4.47	C_15_H_24_N_2_O	[M+H]^+^	249.19613	−1.85	231.1492, 180.0513, 151.9155, 150.0055, 148.0275
	3	isovanillic acid isomers	8.40	C_8_H_8_O_4_	[M-H]^-^	167.03498	−3.53	151.9480, 122.9770, 107.8528
	4	esculetin isomers	11.22	C_9_H_6_O_4_	[M+H]^+^	179.03388	−1.68	160.9808, 150.8389, 134.8390, 132.7981, 122.8295
	5	caffeic acid isomer 2	17.48	C_9_H_8_O_4_	[M-H]^-^	179.03498	−3.46	134.8119, 106.5763
	6	11*β*,13-dihydrolactucin	18.56	C_15_H_18_O_5_	[M+H]^+^	279.12270	−2.65	261.0302, 243.0755, 215.0181, 187.0436
	7	8-deoxylactucin	22.09	C_15_H_16_O_4_	[M+H]^+^	261.11213	−2.07	243.0645, 224.9774, 215.0443, 197.0028, 186.9890
	8 **^a^**	scopoletin	23.32	C_10_H_8_O_4_	[M+H]^+^	193.04953	−1.92	177.9821, 165.0286, 160.9478, 148.9473, 136.9090, 132.8042, 104.9988
	9 **^a^**	quercetin-3-*O*-*β*-D-glucuronide	31.72	C_21_H_18_O_13_	[M-H]^-^	477.06746	−2.26	433.2766, 408.7473, 301.0847, 257.1440, 179.0615
	10 **^a^**	kaempferol-3-*O*-*β*-D-glucuronide	36.27	C_21_H_18_O_12_	[M-H]^-^	461.07254	−3.19	414.8183, 392.8910, 285.0891, 174.8812
	11	isorhamnetin 7-*O*-glucuronide	37.84	C_22_H_20_O_13_	[M-H]^-^	491.08311	−3.10	315.0623, 300.0404, 174.9728
	12 **^a^**	11*β*,13-dihydrolactucopicrin	43.08	C_23_H_24_O_7_	[M+H]^+^	413.15947	−0.56	261.1538, 215.0710
	13	santonin	52.04	C_15_H_18_O_3_	[M+H]^+^	247.13287	−2.10	229.0562, 201.0007, 183.0377, 172.9719, 159.0342
Rats with renal urate deposition	1	esculetin isomers	11.27	C_9_H_6_O_4_	[M+H]^+^	179.03388	0.95	160.8696, 150.8482, 134.8097, 132.8424, 122.9272
	2	11*β*,13-dihydrolactucin	18.52	C_15_H_18_O_5_	[M+H]^+^	279.12270	−1.15	261.0950, 243.0711, 233.1350, 214.9881
	3	8-deoxylactucin	22.05	C_15_H_16_O_4_	[M+H]^+^	261.11213	−2.30	243.1488, 225.0357, 215.0478, 197.0870, 168.9458
	4 **^a^**	scopoletin	23.28	C_10_H_8_O_4_	[M+H]^+^	193.04953	−2.38	177.9432, 164.8572, 160.8682, 136.8060, 132.8942, 104.9909
	5 **^a^**	quercetin-3-*O*-*β*-D-glucuronide	31.70	C_21_H_18_O_13_	[M-H]^-^	477.06746	−1.89	301.0476, 178.9900
	6 **^a^**	kaempferol-3-*O*-*β*-D-glucuronide	36.25	C_21_H_18_O_12_	[M-H]^-^	461.07254	−2.19	285.1616, 175.0296
	7	isorhamnetin 7-*O*-glucuronide	37.82	C_22_H_20_O_13_	[M-H]^-^	491.08311	−3.01	315.1833, 300.1340
	8 **^a^**	11*β*,13-dihydrolactucopicrin	43.09	C_23_H_24_O_7_	[M+H]^+^	413.15947	−2.76	260.9975, 215.0058
	9	santonin	51.99	C_15_H_18_O_3_	[M+H]^+^	247.13287	0.04	229.0214, 201.0340, 183.0294, 172.9458, 158.8941

Note: ^a^ Structures confirmed with chemical standard samples.

**Table 2:** Quantitative RT-qPCR primers and corresponding sequences.

Gene List	Sequence (5′-3′)
*lncRNA H19*	Forward primer: CAGGTAGAGCGAGGTAAAGCA
	Reverse primer: ACACCTGTCATCCTCGCCTT
*ABCG2*	Forward primer: GGCCTGGACAAAGTAGCAGA
	Reverse primer: GTTGTGGGCTCATCCAGGAA
*GAPDH*	Forward primer: GGTGGACCTCATGGCCTACA
	Reverse primer: ATTGTGAGGGAGATCCTCAGTGT
*miR-21-3p*	Stem-loop primer: GTCGTATCCAGTGCAGGGTCC GAGGTATTCGCACTGGATACGACGACAGC
	Forward primer: CGCAACAGCAGTCGATGG
*U6*	Forward primer: CTCGCTTCGGCAGCACA
	Reverse primer: AACGCTTCACGAATTTGCGT

**Table 3:** Database site Information.

Database	Website
BiBiServ2	https://bibiserv.cebitec.uni-bielefeld.de/reputer, accessed on 15 February 2023
lnclocater	http://www.csbio.sjtu.edu.cn/bioinf/lncLocator/, accessed on 15 February 2023
LncATLAS	https://ngdc.cncb.ac.cn/databasecommons/database/id/6018, accessed on 15 February 2023
TargetScan	https://www.targetscan.org/, accessed on 15 February 2023

## Data Availability

The data supporting the findings of this study are available from the corresponding author upon reasonable request.

## Supplementary Materials

The following supporting information can be downloaded at: https://www.mdpi.com/article/10.3390/ijms26167892/s1.

## Abbreviations

ABCG2, ATP-binding cassette subfamily G member 2; ceRNA, competitive endogenous RNA; H&E, hematoxylin and eosin; IL-6, interleukin-6; GLUT9, glucose transporter 9; hs-CRP, hypersensitivity C-reactive protein; lncRNAs, long noncoding RNAs; LPS, Lipopolysaccharide; mRNA, messenger RNA; NRK-52E, Normal rat kidney-52 epithelial cells; ncRNAS, non-coding RNAs; OAT1, organic anion transporters 1; OAT3, organic anion transporters 3; RT-qPCR, reverse transcription quantitative polymerase chain reaction; SCre, Serum creatinine; SUA, serum uric acid; TNF-α, tumor necrosis factor-alpha; UA, uric acid; UCre, urine creatinine; URAT1, urate transporter 1; UUA, urine uric acid.
