# Supplementary material for: Investigation of Pharmacological Mechanisms and Active Ingredients of Cichorium intybus L. in Alleviating Renal Urate Deposition via lncRNA H19/miR-21-3p Regulation to Enhance ABCG2 Expression
Source: Int J Mol Sci. 2025 Aug 15;26(16):7892. doi: 10.3390/ijms26167892 (PMC12386761; doi:10.3390/ijms26167892)
Supplement: Supplementary file 1 [file ijms-26-07892-s001.zip › ijms-3760265-supplementary.pdf]

Supplementary Materials

Investigation of Pharmacological Mechanisms and Active Ingredients of *Cichorium intybus* L. in Alleviating Renal Urate Deposition via lncRNA H19/miR-21-3p Regulation to Enhance ABCG2 Expression

Supplementary Text

Materials and methods

Molecular docking

The 3D structural format files of the active compounds were obtained from the PubChem database (https://pubchem.ncbi.nlm.nih.gov/), where the SDF structure files of the chemical compositions were also retrieved. OpenBabel software was employed to convert these files into the mol2 format. The crystal structure of the ABCG2 protein (PDB ID: 6ffc) was acquired from the RCSB Protein Data Bank. AutoDockTools 1.5.6 software was utilized to perform the dehydration, hydrogenation, and to calculate the Gasteiger charges of the receptor protein. Both ligands and receptors were subsequently converted and maintained in the PDBQT format. AutoDock Vina was used for molecular docking, while PyMOL and PLIP were used for the visualization of results. The database site is shown in **Table S1**.

Table S1 Database site Information

Database	Website
PubChem	https://pubchem.ncbi.nlm.nih.gov/
RCSB Protein Data Bank	https://www.rcsb.org/
PLIP	https://plip-tool.biotec.tu-dresden.de/plip-web/plip/index

Supplementary Figures

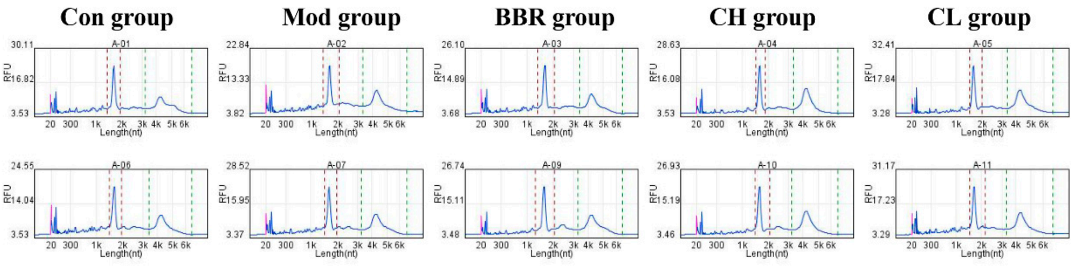

Fig. S1. Representative images demonstrating the integrity of total RNA.

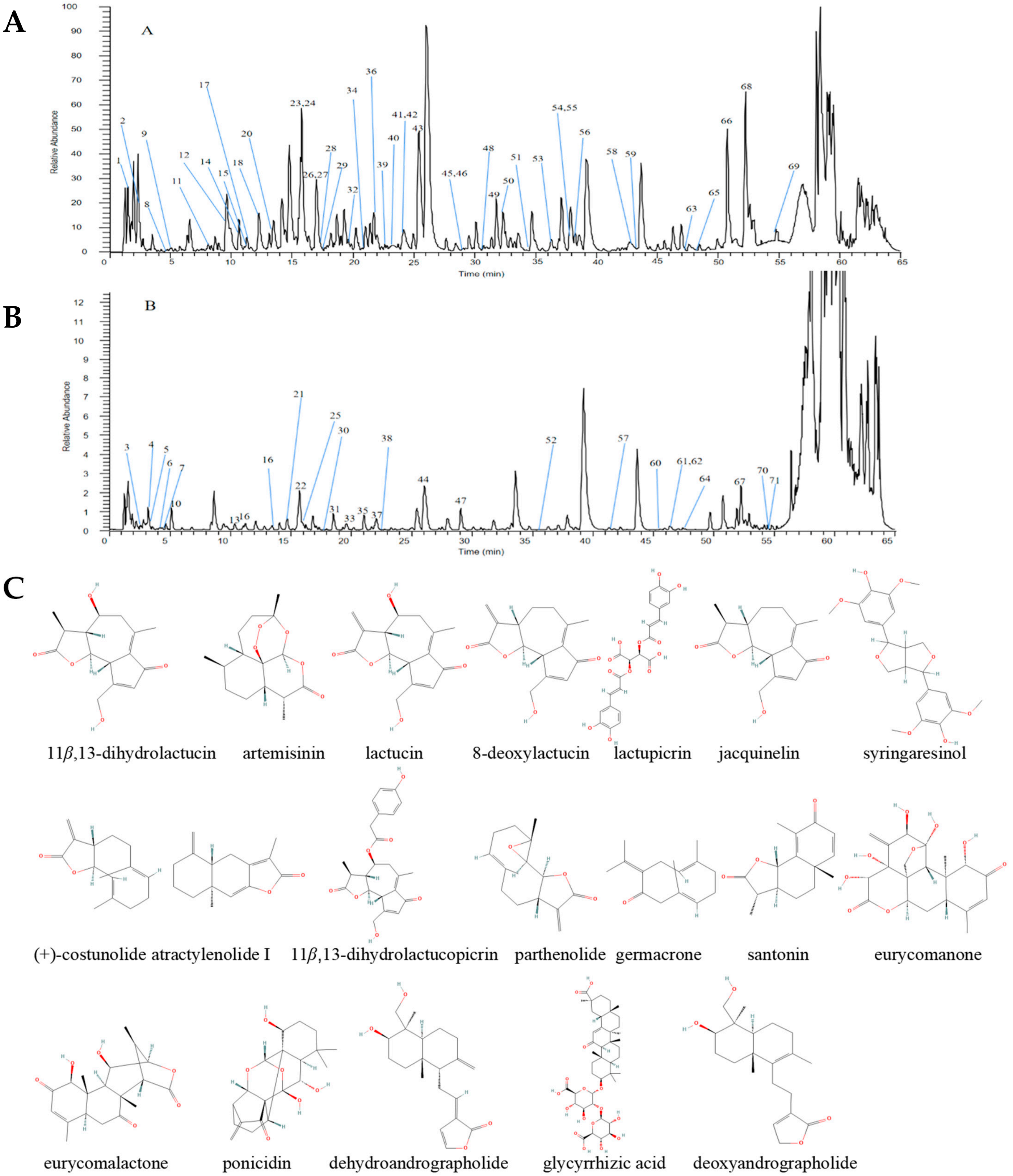
A
Relative Abundance
Time (min)
B
Relative Abundance
Time (min)
C
11β,13-dihydrolactucin
artemisinin
lactucin
8-deoxylactucin
lactupicrin
jacquinelin
syringaresinol
(+)-costunolide
atractylenolide I
11β,13-dihydrolactucopicrin
parthenolide
germacrone
santonin
eurycomanone
eurycomalactone
ponicidin
dehydroandrographolide
glycyrrhizic acid
deoxyandrographolide

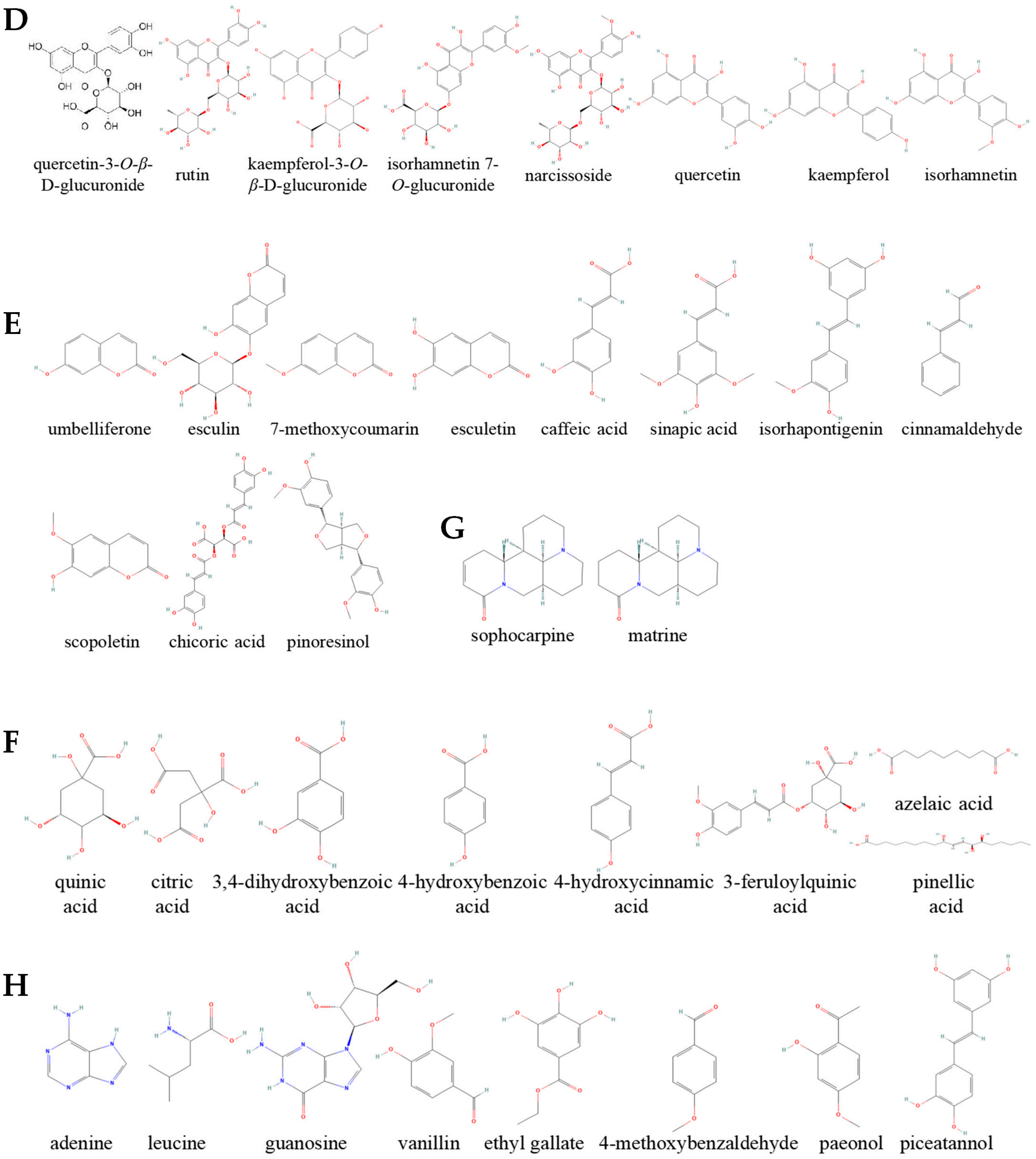

Fig. S2. Chemical analysis of chicory extract. Ultra performance liquid chromatography total ion chromatogram of chicory extract in (A) negative and (B) positive ion modes. (C) Terpenoids identified

in chicory extract. (D) Flavonoids identified in chicory extract. (E) Phenylpropanoids identified in chicory extract. (F) Organic Acids identified in chicory extract. (G) Alkaloids identified in chicory extract. (H) other compounds identified in chicory extract.

A

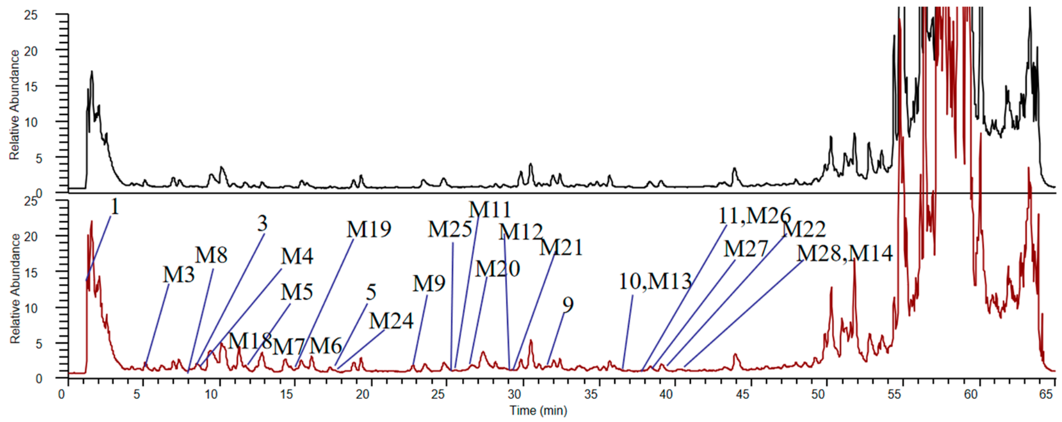

B

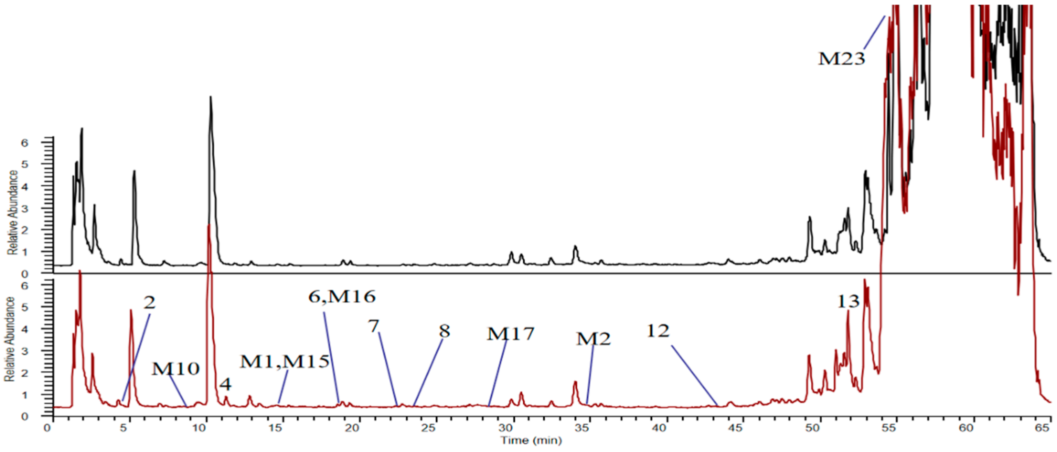

C

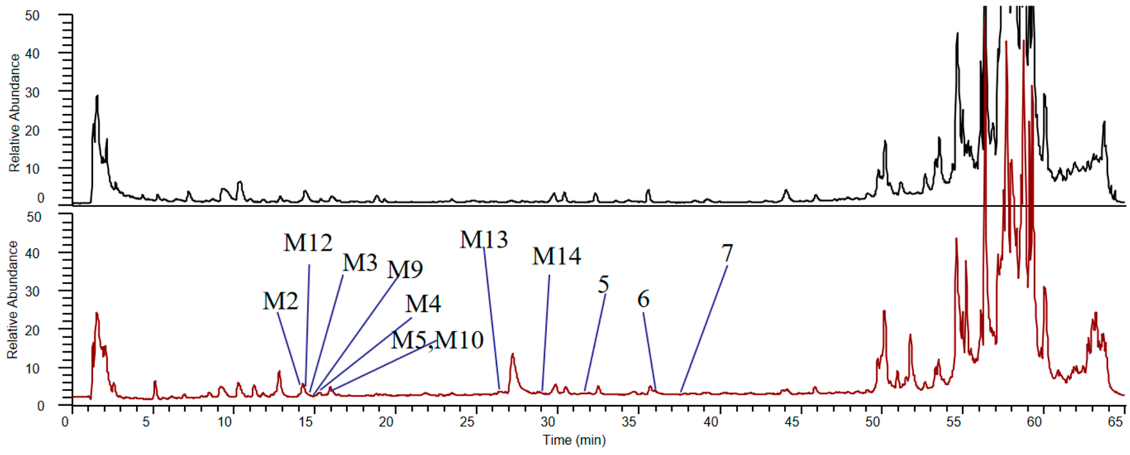

D

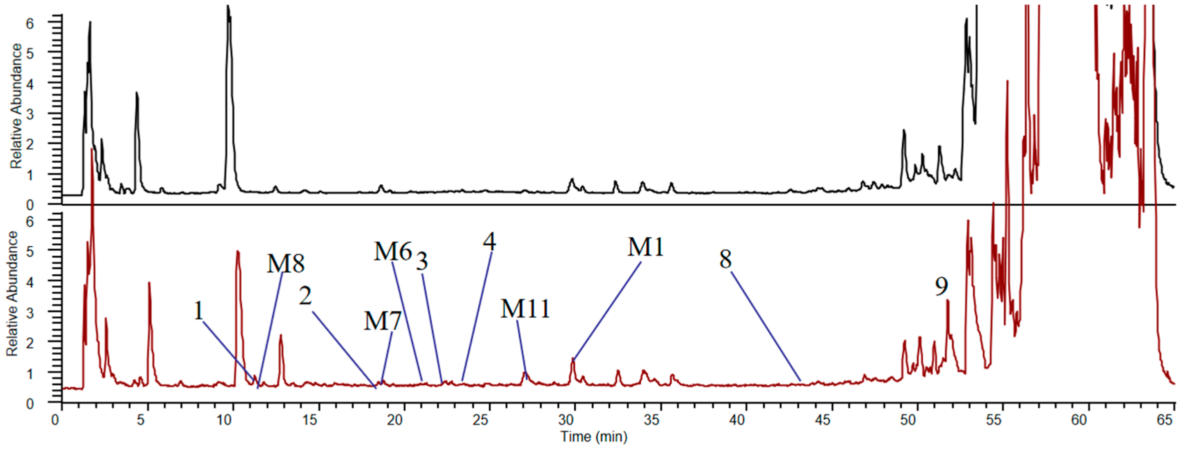

Fig. S3. Total ion chromatograms of plasma samples. (A) & (B): Total ion current maps of blank plasma (upper panel) and chicory-containing plasma (lower panel) from normal rats, under negative ion mode (A) and positive ion mode (B), respectively. (C) & (D): Total ion current maps of blank plasma (upper panel) and chicory containing plasma (lower panel) from rats with renal urate deposition, under negative ion mode (C) and positive ion mode (D), respectively.

(**A**) Molecular docking of 11*β*,13-dihydrolactucopicrin with ABCG2 protein

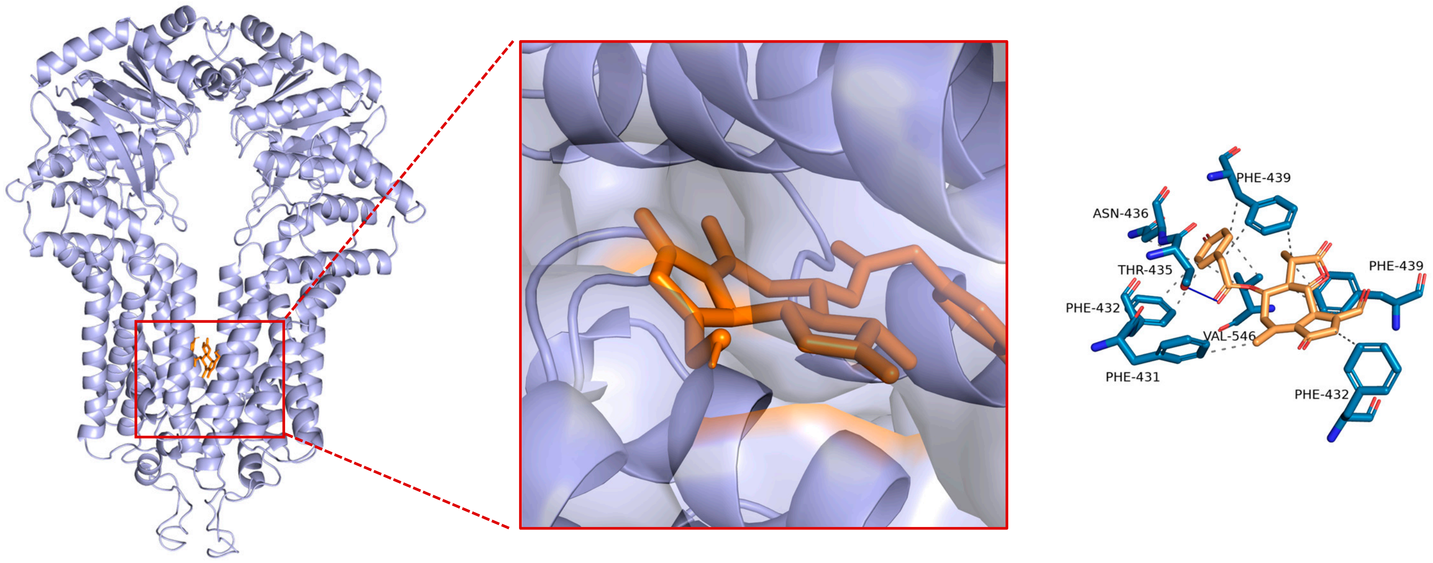

(B) Molecular docking of **quercetin-3-*O*-*β*-D-glucuronide** with ABCG2 protein

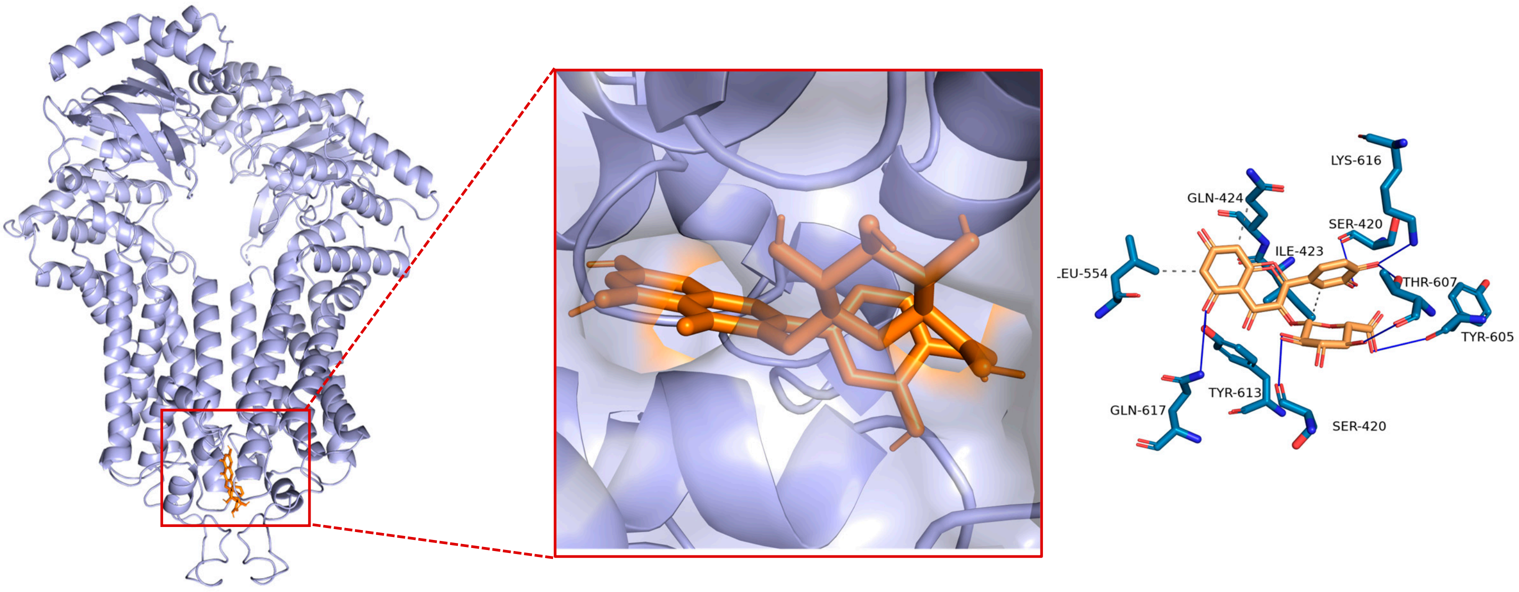

(C) Molecular docking of **kaempferol-3-*O*-*β*-D-glucuronide** with ABCG2 protein

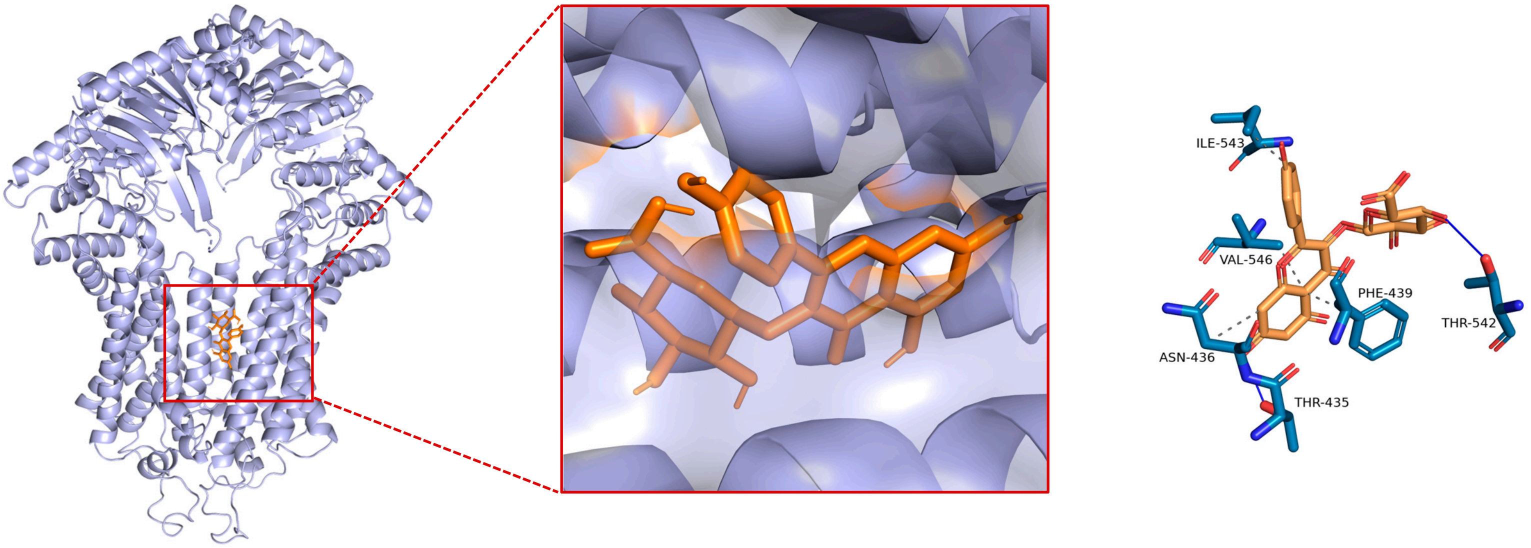

(**D**) Molecular docking of scopoletin with ABCG2 protein

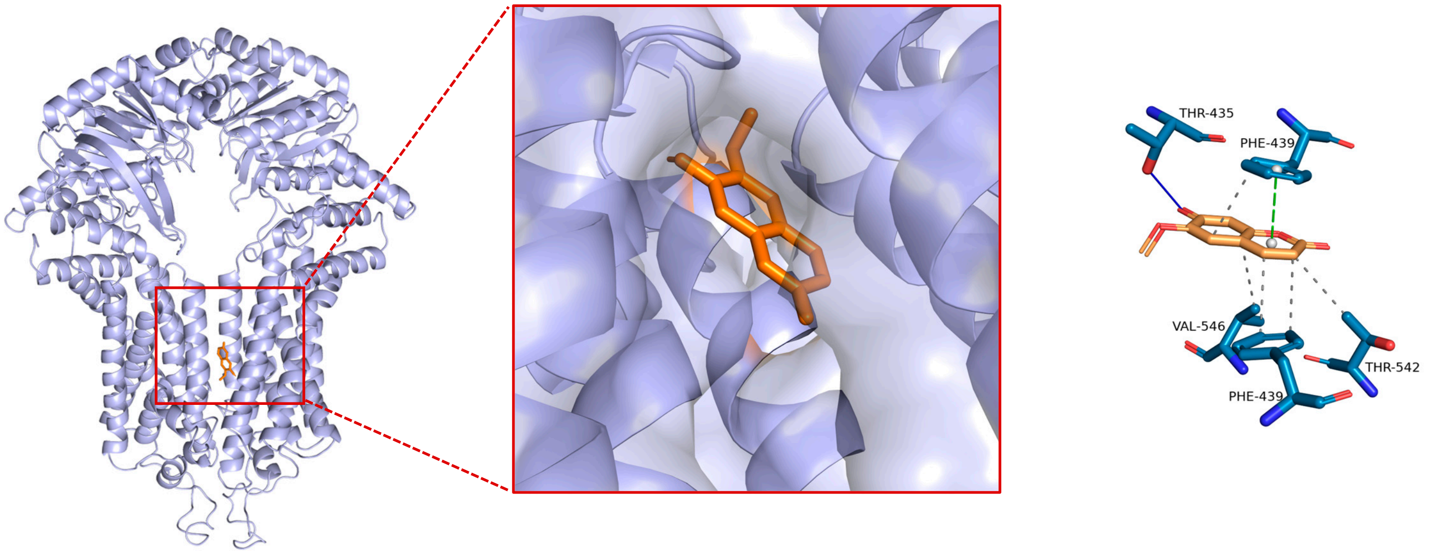

Fig. S4. Molecular docking modes of four chemical components with ABCG2 protein. **(A)** Molecular docking of 11*β*,13-dihydrolactucopicrin with ABCG2 protein. **(B)** Molecular docking of quercetin-3-*O*-*β*-D-glucuronide with ABCG2 protein. **(C)** Molecular docking of kaempferol-3-*O*-*β*-D-glucuronide with ABCG2 protein. **(D)** Molecular docking of scopoletin with ABCG2 protein.

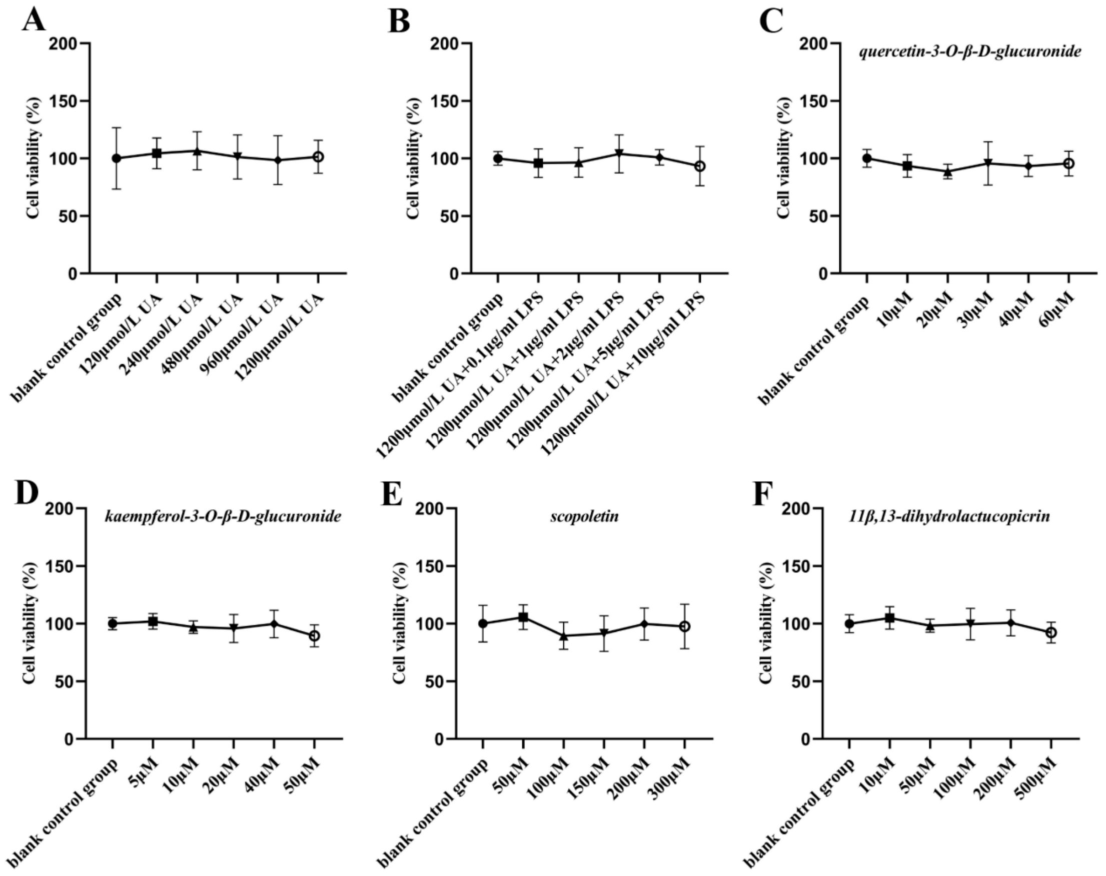

Fig. S5. Determination of cell viability of modeling agents and chemical components. **(A)** Cell viability of NRK-52E cells treated with varying concentrations of UA (120/240/480/960/1200 μmol/L) (*n*=6). **(B)** Cell viability of NRK-52E cells treated with 1200 μmol/L UA and different concentrations of LPS (0.1/1/2/5/10 μg/mL) (*n*=6). **(F-I)** Effects of different concentrations of quercetin-3-*O*-*β*-D-glucuronide or kaempferol-3-*O*-*β*-D-glucuronide or scopoletin or 11*β*,13-dihydrolactucopicrin on the cell viability of NRK-52E cells treated with 1200μmol/L UA and 1 μg/mL LPS (*n*=6). $^{*}p < 0.05$, $^{**}p < 0.01$, compared with the UA and LPS-treated group.

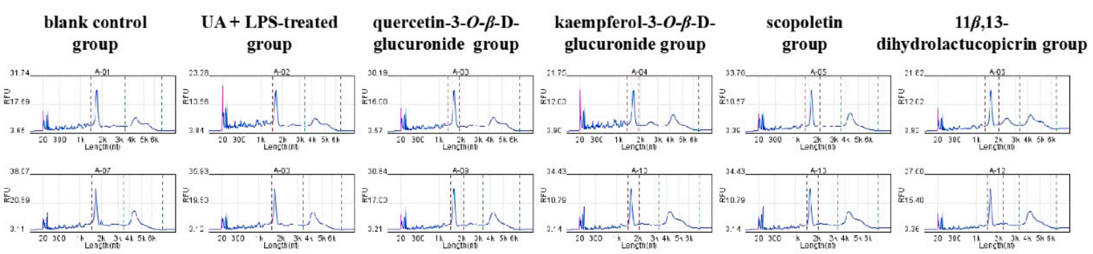

Fig. S6. Representative images of total RNA integrity.

Table S2 Free binding energies of four active ingredients binding to ABCG2

No.	Compound	Target	Binding energy (kcal/mol)
1	11*β*,13-dihydrolactucopicrin	ABCG2	-11.2
2	quercetin-3-*O*-*β*-D-glucuronide		-9.9
3	kaempferol-3-*O*-*β*-D-glucuronide		-8.5
4	scopoletin		-7.5

Table S3 Structure-Activity Relationship between four chemical components and ABCG2

No.	Interacting group	Hydrophobic Interactions	Hydrogen bond	π-π stacking	Salt bridge
1	11*β*,13-dihydrolactucopicrin	PHE-431B,PHE-432A, PHE-432B,ASN-436B, PHE-439A,PHE-439B, 38VAL-546A	THR-435B	/	/
2	quercetin-3-*O*-*β*-D-glucuronide	ILE-423A,GLN-424A, LEU-554A	SER-420A,SER-420B, TYR-605B,THR-607B, TYR-613A,LYS-616B, GLN-617A	/	/
3	kaempferol-3-*O*-*β*-D-glucuronide	ASN-436B,PHE-439B, ILE-543A,VAL-546A	THR-435B,THR-542B	/	/
4	scopoletin	PHE-439A,PHE-439B, THR-542B,VAL-546B	THR-435A	PHE-439A	/
